# Video Q&A: State-of-the-art therapy for the elite and non-elite athlete: an interview with Mike Carmont

**DOI:** 10.1186/1741-7015-12-8

**Published:** 2014-01-17

**Authors:** Michael R Carmont

**Affiliations:** 1 Princess Royal Hospital, Telford, UK

## Abstract

In this video Q&A, Mr Mike Carmont answers questions about state-of-the-art treatments for elite athletes, and the progress and challenges behind translating these into successful therapies for the non-elite athlete.

## Mike Carmont discusses state-of-the-art therapy for the elite and non-elite athlete

## Introduction

I am a consultant trauma and orthopaedic surgeon with an interest in sports medicine. I think, looking back, my first interest in sports medicine started when I submitted a presentation to the first Consensus Meeting for Concussion in Sport in Zurich - that was over 12 years ago. I was attracted to the demonstration of the interaction of science, medicine and sport. After that, I went to the 2002 Commonwealth Games where I was an event-side doctor at the field of play and saw some sports practice in action. Since then, I’ve been attracted to the precision and accuracy of the sports surgery techniques throughout my orthopaedic training, and that’s something that I continue in my current practice. I am currently the President of the British Orthopaedic Sports Trauma and Arthroscopy Association.

**Figure:**
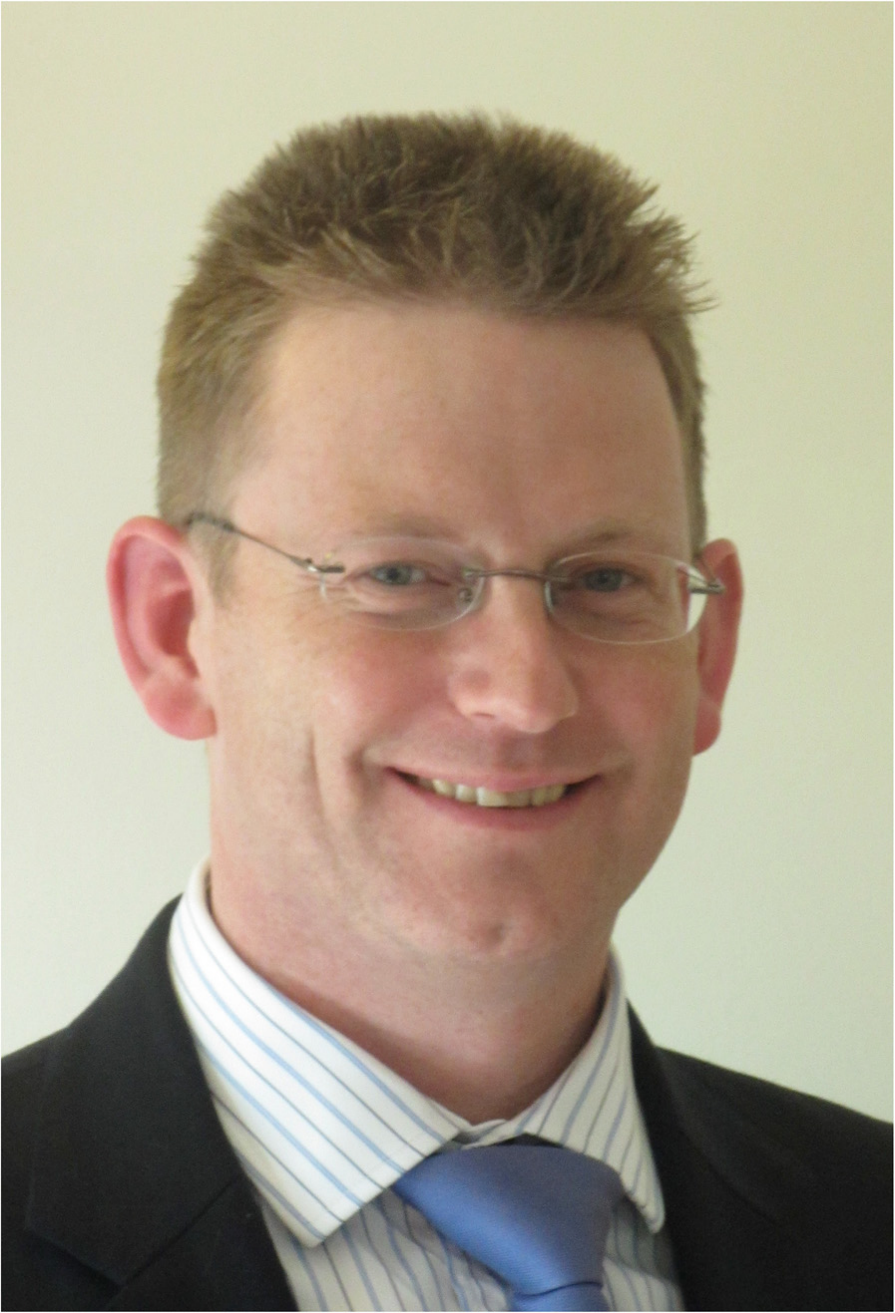
Mike Carmont is a consultant trauma and orthopaedic surgeon based at the Princess Royal Hospital, Telford.

## Transcript

### 1. As an orthopaedics surgeon, does your treatment of athletes differ from your treatment of those not involved in sports?

Not really. All the patients I treat are treated the same way. I try to use the best evidence I have from the literature and I discuss the treatment options of any particular problem. We have to remember that the patients themselves choose which procedure they wish to have performed, and they need to understand the outcomes of the treatment selected. A typical case of this would be meniscal injury. Many years ago, it was thought the meniscus was a vestigial structure in the knee, and following a tear, these would just be removed. But now we know the importance of the meniscus for loading, stability and proprioception, and nutrition within the knee. We now know that a meniscectomy does lead to arthritis. Meniscal repair is not 100% successful, but in the majority of cases, it does lead to a good recovery. That allows our patients to return to sports and activity with less of a risk of the onset of arthritis as a result of their tear.

### 2. When treating elite athletes, do you ever have to compromise long-term health outcomes for short-term sports performance gains?

No, I try as best I can to manage all my patients the same. I try and promote physical activity for all patients. It’s very important when managing the social, recreational and elite or professional athletes. I try and use standard, best evidence and well-researched techniques where possible to improve outcome.

### 3. In terms of surgery for the athlete, have there been any advances within the past few years that could potentially allow that athlete to return to the same form pre-injury?

Anterior cruciate ligament (ACL) injuries are one of the most common knee injuries. It tends to occur in a young, sporting population during sport, particularly ones that involve a cut or plant, or a sudden change in direction, particularly in popular sports such as football and skiing. Whereas people with these injuries may be able to adopt normal activities of daily living, the absence or tear of this ligament mean that they may not be able to control their knee in these manoeuvres, and as a consequence give up these sports.

The surgery for ACL is fascinating. The techniques have evolved over the past two decades. Whereas originally surgeons were performing trans-tibial techniques to try and replace and reconstruct the ligament across the knee, now they are trying to restore a more natural and anatomical appearance to the reconstruction, with the use of double-bundle and more anatomical single-bundle techniques. This is one of the key components of sports surgery. One has to ascertain whether these techniques themselves are an improvement - that is one of the significant challenges. We need to have very accurate outcome scores and assessments, and it could be that the small improvements that are made are not determined and evaluated by the outcome measurements we have. For instance, for the more anatomical single-bundle ACL reconstructions, some groups have suggested that these may be more prone to tearing and have a higher re-rupture rate. This could be because the knee is more restrained and adopts a more natural position, and that the reconstructed tissue has a greater load than the tissue placed elsewhere in the knee.

### 4. Are there any advantages in ACL repair versus ACL reconstruction?

That’s another interesting development. Over the past few years, people have reflected back to the consideration of repairing the torn ACL, rather than reconstructing it. Here, the body has its own tissue put back in the natural place. But we have to remember that this tissue has strained and then ultimately failed before reattachment, and so the procedure is very difficult to do. With the use of scaffolds and matrices put inside the knee, this is something that will become increasingly common in the next decade. Science will show us if this is an improvement or not.

### 5. Have there been any advances in sports surgery in the elite athlete that have translated into treatment options for the non-athlete and recreational athlete. For example, with platelet-rich plasma?

Platelet-rich plasma (PRP) is the concept where you take someone’s whole blood, place it into a centrifuge, and spin it out into it component products. You can then take a small amount of the serum which is rich in growth factors, which has biological factors to promote healing and enhance recovery. You’d then re-inject this into the athlete into an area of, for example, degenerative tissue. Classic problems for this would be tendinopathy, or enthesopathies such as lateral epicondylitis, and also Achilles tendinopathy. These are very difficult problems to treat and they are also reasonably common in the general population.

### 6. Are there any challenges in comparing treatments tested in elite athletes for translation into the non-athlete?

Elite athletes are a difficult group to compare - they’ll use any technique to get them back to return to play. This may well mean that, in terms of their overall return to activity, they are harder to follow and not directly comparable to the general population. This is because the elite athlete will have better physiotherapy, treatment modalities and better rehabilitation techniques, and also better motivation, too. So the outcomes in elite athletes are possibly not comparable to the general population.

### 7. What is the current evidence supporting the use of PRP?

There have been a number of studies which have looked at these, and it’s very hard to show that the administration of these techniques shows a definite difference in a randomized prospective study.

For instance, at the elbow, Mishra from California has reported significant improvement in visual analogue scores for pain (*P* = 0.02); [at 24 weeks with PRP in a double-blind prospective randomized study on 230 patients with chronic tennis elbow]. However, when compared in another group from Denmark using a randomized study of PRP and glucocorticoids, they showed no significant benefits of any of the treatment arms of the PRP compared to the placebo arms.

Achilles tendinopathy is another common problem. Susan de Jonge’s group in the Netherlands have put a lot of work into this, and they found that tendinopathy is very common; between two and three of every 1,000 members of the Dutch population will have Achilles tendinopathy. It is a very troublesome problem - it’s chronic. The same research group have looked at the overall outcome, and five years after all treatments, 40% of people will still have levels of pain and dysfunction. Yes, their symptoms may have improved, but they will no longer be pain-free as a result of their problem.

Various studies have looked at the use of PRP versus the use of placebo concurrently with eccentric loading exercises, and these haven’t shown a significant benefit. The tendon structure has improved and you get a more natural appearance of the tendon, and it’s thought that it’s the loading exercises which are achieving this. Conversely, across the Atlantic, Owens’ group showed a modest improvement. So, it can be very difficult to tell if it is the PRP which is making a difference, which is why we do need to have randomized and prospective controlled studies to try and resolve these issues.

### 8. Where can I find out more?

See references [1-15].

## Abbreviations

ACL: Anterior cruciate ligament; PRP: Platelet-rich plasma.

## Competing interests

The author declares he has no competing interests.

## Pre-publication history

The pre-publication history for this paper can be accessed here:

http://www.biomedcentral.com/1741-7015/12/8/prepub

## Supplementary Material

Mike Carmont discusses state-of-the-art therapy for the elite and non-elite athlete Click here for file
